# Temporal and Spatial Properties of Arterial Pulsation Measurement Using Pressure Sensor Array

**DOI:** 10.1155/2012/745127

**Published:** 2011-06-30

**Authors:** Chung-Shing Hu, Yu-Feng Chung, Cheng-Chang Yeh, Ching-Hsing Luo

**Affiliations:** ^1^Department of Electrical Engineering, National Cheng Kung University, Tainan 70101, Taiwan; ^2^Nanjing University of Chinese Medicine, Nanjing 210046, China

## Abstract

Conventionally, a pulse taking platform is based on a single sensor, which initiates a feasible method of quantitative pulse diagnosis. The aim of this paper is to implement a pulse taking platform with a tactile array sensor. Three-dimensional wrist pulse signals are constructed, and the length, width, ascending slope, and descending slope are defined following the surface of the wrist pulse. And the pressure waveform of the wrist pulse obtained through proposed pulse-taking platform has the same performance as the single sensor. Finally, the results of a paired samples *t*-test reveal that the repeatability of the proposal platform is consistent with clinical experience. On the other hand, the results of ANOVA indicate that differences exist among different pulse taking depths, and this result is consistent with clinical experience in traditional Chinese medicine pulse diagnosis (TCMPD). Hence, the proposed pulse taking platform with an array sensor is feasible for quantification in TCMPD.

## 1. Introduction

There are four diagnostic methods, namely, inspection, listening and smelling, inquiry, and palpation, in traditional Chinese medicine (TCM) to diagnose causes of disease, locations of disease, the nature of disease, and predictions of a cure [1]. This means that palpation reflects the health condition of patients. If TCM physicians are good at pulse diagnosis, then they will have a good command of detailed changes in diseases. Following clinical evidence, pulse diagnosis builds an unshakable position in TCM. However, it requires long-term experience and a high level of skill for doctors to master pulse diagnosis. Researchers have done many reports to shorten the training duration and to enhance effective diagnosis using pulse diagnosis by means of modern technology [2–5]. The first step is to acquire the wrist pulse by using a sensor.

From the viewpoint of hemodynamical theories, Wang et al. pointed out the pulse wave should be governed by both a longitudinal and a transverse wave model [6, 7]. Hence, the investigation of a pulse wave has to include both temporal and spatial dimensions. TCM physicians use a finger to take physiological information from a wrist radial artery; this technique is called *pulse diagnosis* or *palpation,* since the feeling of a pulse wave is acquired from the surface of the finger. Hence, the analysis of a wrist radial artery also has to include both temporal and spatial dimensions. From the above discussion, it can be seen that a pulse taking platform with an array sensor is needed.

Therefore, sensor research has shifted a single sensor to an array sensor or multiple combined sensors to obtain more information about wrist pulse signals [8–14]. Strain gauge, piezoresistor, and polyvinylidene fluoride (PVDF) are common choices for obtaining wrist pulse signals. The sensitivity, spatial resolution, and sensing area of a sensor are critical issues in the modernization of TCM. The optimal sensing area of a single sensor is about 30 mm^2^ [5]. However, the sensing area of the sensing element is too large to obtain a good spatial resolution for a sensor array. To increase the spatial resolution, the sensing element has to decrease the sensing area but at the same time guarantee that the sensitivity is great enough (about 25 mmHg) [15].

**Figure 1:**
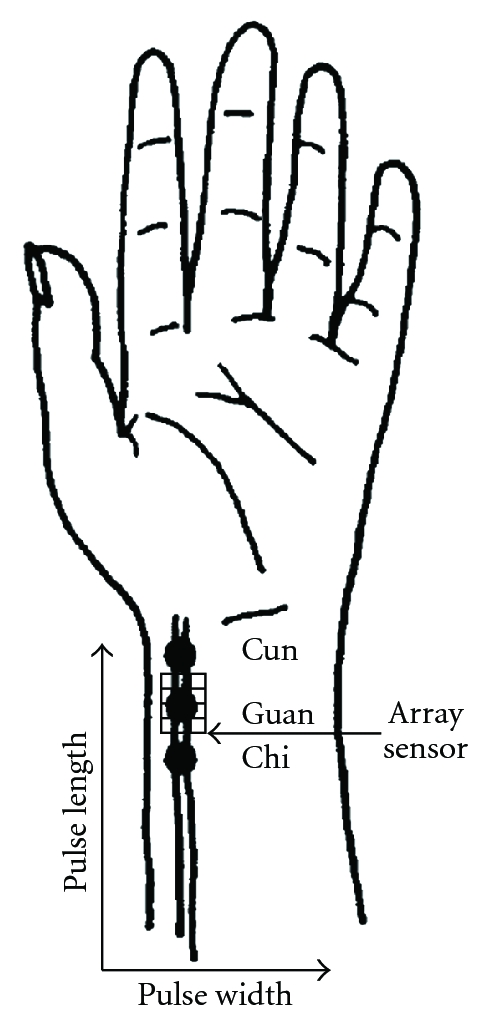
A diagram of the implementation of the pulse taking platform with an array sensor to measure the length and width of wrist pulse signals.

Hence, we design the pulse taking platform with a tactile capacitive array sensor to provide quantifiable TCMPD research. The pre-experiments reveal that the wrist radial artery waveform from one sensing element of the tactile capacitive array sensor was the same as the proposed pulse taking platform with a single sensor. Based on this result, we assume that a pulse taking platform with a tactile capacitive array sensor is feasible for quantifiable TCMPD research and will obtain more information than the proposed pulse taking platform with a single sensor. The temporal and spatial properties of the wrist radial artery are illustrated, including strength, rate, length, width, and trends of pulse conditions, as shown in Figure 1. In Figure 1, pulse length represents the sensing length of physician's feeling during the pulse taking procedure. Similarly, pulse width represents the sensing width of this procedure. In addition, the hold-down pressure is provided by the proposed array sensor.

The results of the experiments match that of clinical experiences. Therefore, a pulse taking platform with a tactile capacitive array sensor is feasible for quantifiable TCMPD research.

## 2. Materials and Methods

### 2.1. Sensor Principle

**Figure 2:**
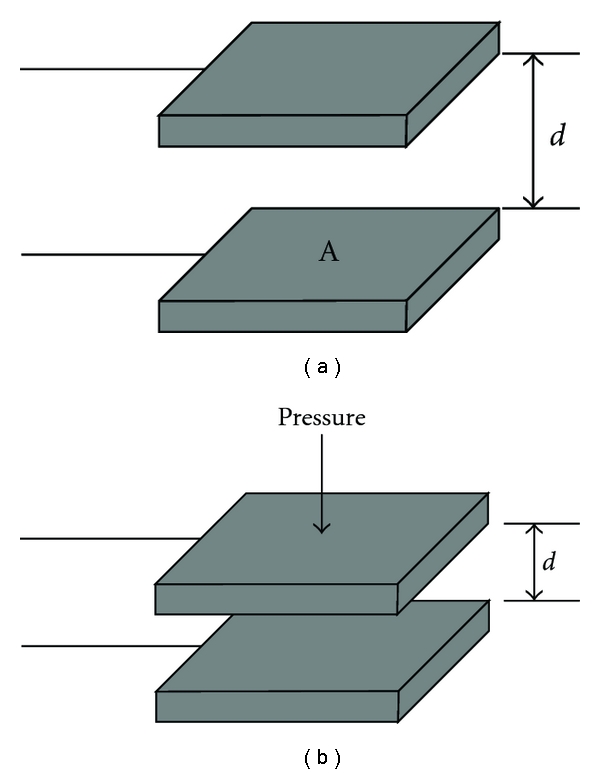
(a) The structure of a parallel plate capacitor. (b) The distance of the gap decreases and the capacitance goes up in response to the increase of the pressure.

 The main specifications of the sensor in our proposal are a sensitivity of about 25 mmHg (or 0.48 psi) and a sensing element area of about 10 mm^2^. Under these conditions, the technology of the pressure profile system company (PPS) meets our requirements. A brief description about the sensor technology is addressed as below. The capacitance can be calculated if the geometry of the conductors and the dielectric properties of the insulator between the conductors are known. For example, the capacitance of a parallel-plate capacitor is composed of two parallel plates with area *A* separated by a distance *d* that is approximately equal to the following:

(1) $$C=\varepsilon \frac{A}{d},$$

where *C* is the capacitance, *A* is the area of the overlapping of the two plates, *ε* is the dielectric constant, and *d* is the separation between the plates as shown in Figure 2(a). 

**Figure 3:**
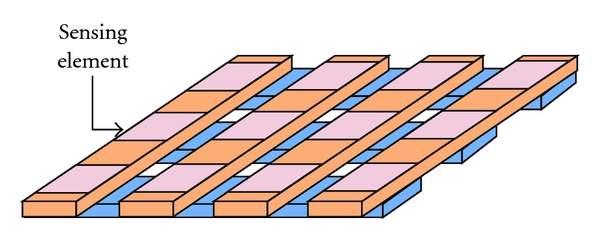
To built the array sensor, the electrodes can be arranged orthogonally. Each capacitor is located at each point where the electrodes overlap.

**Figure 4:**
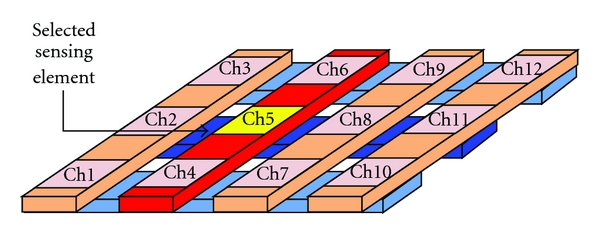
The capacitor is selected by the number of row and column to measure the pressure waveform at the intersection point.

 If the separation distance decreases, the capacitance *C* goes up as shown in Figure 2(b). When building tactile array sensors, the electrodes can be arranged as orthogonal, overlapping strips. A distinct capacitor is formed at each point where the electrodes overlap, as shown in Figure 3. By selectively scanning a single row and column, the capacitance at that location, as well as the local pressure is measured, as shown in Figure 4. Therefore, the wrist pulse signals can be detected based on capacitive tactile sensors. 

The specifications of the tactile array sensor for this proposed pulse taking platform are an array size of 10 mm × 7.5 mm, a sensing element of 2.5 mm × 2.5 mm, a thickness of approximately 0.5 mm, a full-scale range of 300 mmHg, sensitivity of 0.5 mmHg, a scan rate of 100 Hz, a temperature range of −20 to 100°C. The capacitive tactile sensor is custom designed by PPS, USA. 

### 2.2. Pulse Taking Platform

**Figure 5:**
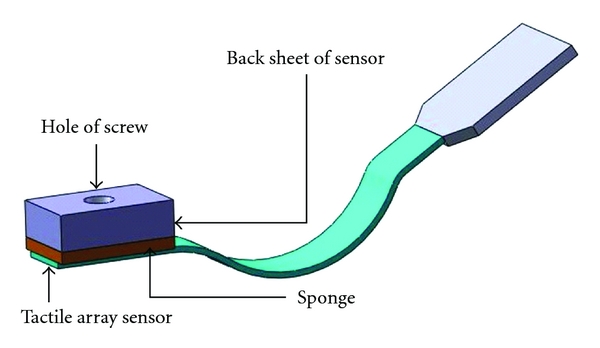
A display of the structure of a tactile array sensor block. The tactile array sensor includes twelve sensing points.

 Conventionally, the pulse taking platform has used a single sensor, but the information obtained could not be compared with the real feeling of the physician's fingertip. Hence, a modified pulse taking platform with the sensor array is implemented to acquire detailed information regarding wrist pulse signals. It can simultaneously detect twelve channel signals at one sensing position, such as Cun, Guan, or Chi. The sensor block is flexible, and its structure is displayed in Figure 5.

**Figure 6:**
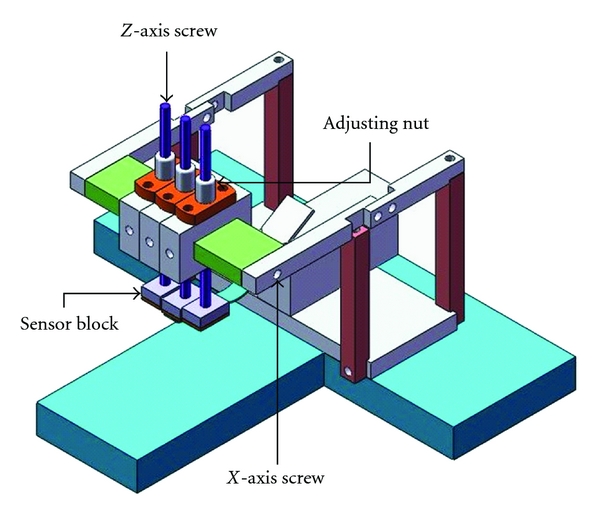
The proposed two-axis pulse taking platform. The nut and *X*-axis screw can be adjusted to mimic the pulse taking procedure of physicians.

In addition, the pulse taking platform, which is employed to determine the best measurement points on the *X* and *Z* axes, is adjustable with regard to the *X* and *Z* axes. Three screws and sleeves are used in the *Z*-axis to produce movement to hold down the tactile array sensor block. To obtain an acceptable pulse-taking position, a rotating device is housed in the *X*-axis for adjustment. The proposed two-axis pulse taking platform is shown in Figure 6. Additionally, the acquisition of wrist pulse signals uses an analog-to-digital card (D600, PPS, USA). Its sampling frequency is 100 Hz; each sensing element is calibrated by software.

### 2.3. Data Collection

 This study attempts to minimize variations resulting from gender and health conditions. The subjects are all males (average age of 20.64 ± 6.84) who have no diseases, as confirmed by a TCM physician. The procedures for the experiment were approved by the Air Force Academy and National Cheng Kung University. The experimental sample consists of five R.O.C Air Force Academy students and one lab student.

 The goal of this study is to investigate the feasibility of a pulse taking platform with a sensor array. Based on TCM clinical experiences with pulse diagnosis, the pulse conditions are not expected to change during a 10-minute period of time. For the sampling facility, firstly, this study chooses one volunteer and records his wrist pulse signals twice in 10 minutes and checks whether the pulse signals are same or not. At the time of each sampling, the volunteer was asked to stop exercising and to rest for 5 minutes before the pulse conditions were sampled. During the pulse condition sampling, the subject was asked to sit on an adjustable chair, and he was forbidden to move his arterial wrist. Each sampled pulse was taken by pressing with only one finger, that is, only one robotic finger taking the pulse at Guan. In this study, only Guan data is analyzed. One of the reasons for this is that the strength of the wrist pulse at Guan is generally stronger than other pulse taking positions. First, the physician carries out the pulse taking and marks the Cun, Guan, and Chi positions. Then, the participant places the marked position of the arterial wrist under the sensor block, adjusts the screw about 5 times (from lightly touching skin to the bone), the depth of each time is 0.5 mm, and then samples the wrist pulse at this depth. The number of sampling data totaled 20. Each acquisition time was about 15 seconds.

The second experiment is using the proposed pulse taking platform to evaluate the differences of pulse conditions among pulse taking depths, such as Fu, Zhong, and Chen, the wrist pulse signals are similarly sampled as in the above method. The participants place the marked position of the arterial wrist under the sensor block, and the operator adjusts the screw about 12–18 times (from lightly touching skin to the bone), the depth of each time is 0.5 mm, and samples the wrist pulse at each depth. According to these data, the pulse taking platform performance suggested in this proposal is derived for later evaluation.

### 2.4. Signal Analysis

**Figure 7:**
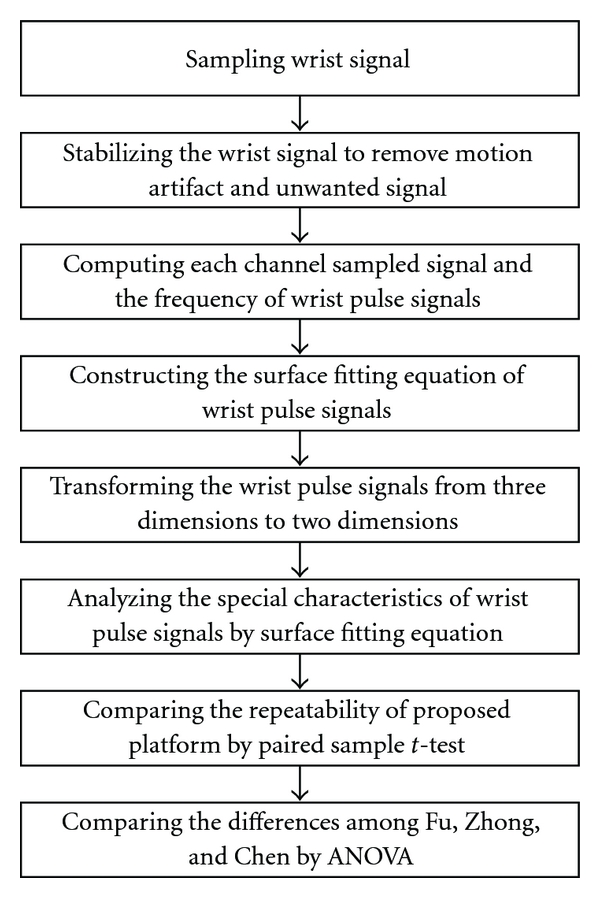
A flowchart showing the signal processing procedure for the study.

**Figure 8:**
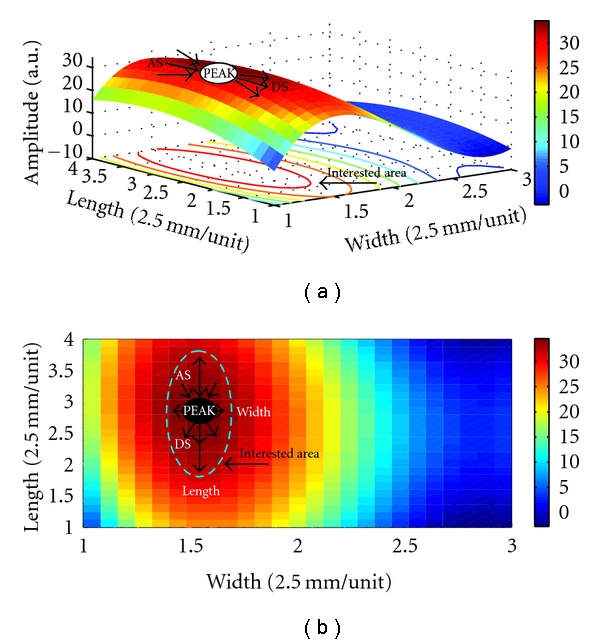
(a) The surface wrist pulse is constructed by polynomial fitting equation. (b) The parameters LENGTH, WIDTH, AS, and DS are defined using an *X*-*Y* projection.

A wavelet algorithm is adopted to remove baseline wander and high-frequency noise [16], and then, a polynomial surface fitting is adopted to fit these processed signals. The detailed information for the signal processing is displayed in Figure 7. The surface of the wrist pulse is constructed based on fitting the equation, as shown in Figure 8(a). 

**Figure 9:**
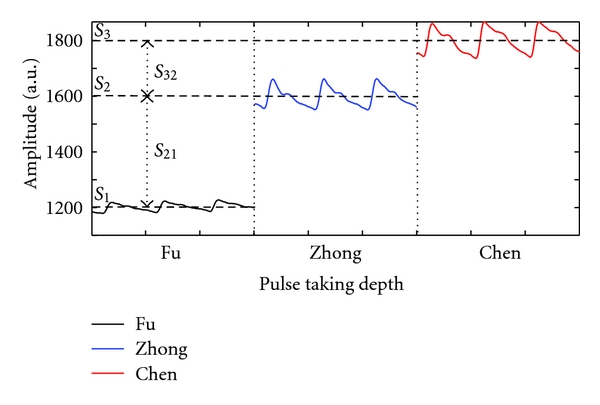
The pulse waveforms are detected from the same channel but at different pulse taking depth. The horizontal dash line represents the static pressure at each pulse taking depth.

The surface of wrist pulse is projected to an *X*-*Y* plane. *X* indicates the width of wrist pulse, and *Y* is the length of the wrist pulse. Some core characteristics such as PEAK, FREQ, LENGTH, WIDTH AS, DS, and STATIC are defined as parameters for analysis later. PEAK is the peak of the surface wrist pulse. Once the PEAK is known, the area of interest is also determined through our experimental process. FREQ is the frequency of a wrist pulse defined in the maximum peak to peak for the sampling channel at each pulse taking depth. LENGTH, WIDTH, AS, and DS are defined at the peak of the surface wrist pulse at each pulse taking depth shown in Figure 8(b): LENGTH, the length of the wrist pulse according to the area of interest along the *Y*-axis; WIDTH, the width of the wrist pulse along the *X*-axis; AS, the ascending slope (AS) around the peak point of the surface wrist pulse; DS, the descending slope (DS). STATIC is the direct current component of the wrist pulse signals, which represents the hold-down pressure or static pressure, shown in Figure 9. ΔSTATIC, such as S_21_ and S_32_, represents the differences in STATIC between pulse taking depths.

### 2.5. Statistical Method

According to the above defined parameters, this study intends to check the repeatability of the proposed pulse taking platform and the differences of pulse conditions among Fu, Zhong, and Chen pulse-taking depths. To acquire a statistical analysis, we use the SPSS 17.0 program. To guarantee the repeatability of the proposed pulse taking platform in order to satisfy the requirements of pulse diagnosis, a paired samples *t*-test was carried out.

Additionally, a one-way analysis of variance (ANOVA), STATIC, PEAK, FREQ, LENGTH, WIDTH, AS, and DS of the wrist pulse at each pulse taking depth of Fu, Zhong and Chen are examined. Their mean differences between pulse taking depths are also verified, and Scheffe's test and Tamhane's test are carried out for multiple comparisons.

## 3. Results

### 3.1. Results of the Proposed Pulse Taking Platform

**Figure 10:**
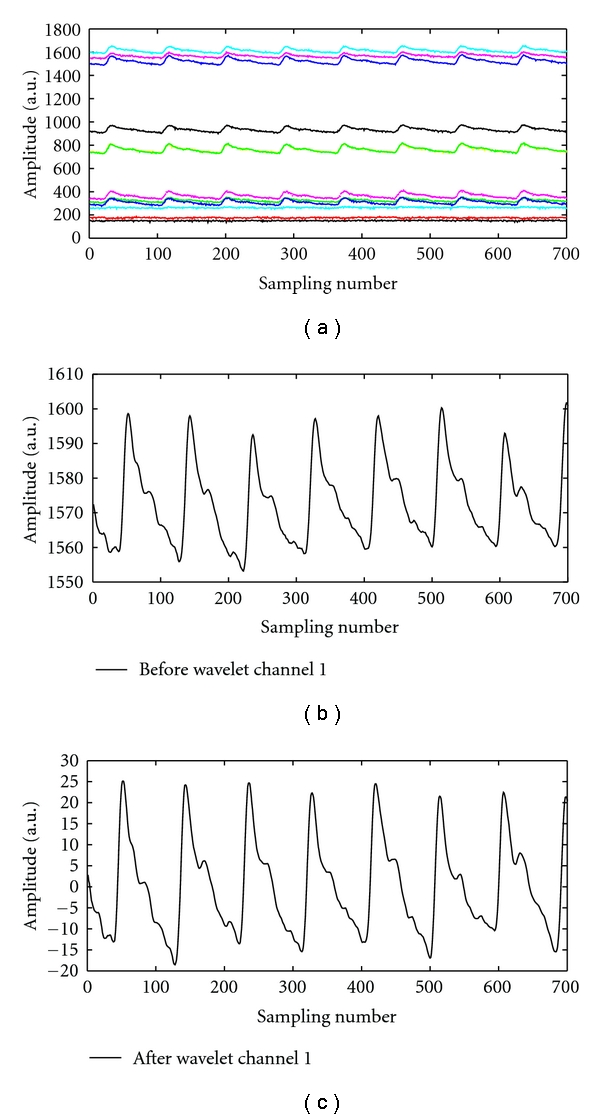
Pressure waveforms measured by the proposed pulse taking platform: (a) 12-channel raw data of wrist pulse signals, including pulse waveform and hold-down pressure. (b) The wrist pulse signals before the wavelet algorithm. (c) The wrist pulse signals after the wavelet algorithm.

The proposed pulse taking platform detected the wrist pulse signals as shown in Figures 9 and 10.  Figure 10(a) shows the original sampled signal; each channel presents dynamic wrist pulse signals on the static pressure or the hold-down pressure. Figures 10(b) and 10(c) indicate the original signals (Figure 10(b)) at one of 12 channels and the signals (Figure 10(c)) processed by the wavelet algorithm to remove the static pressure and baseline drift. It can be seen that the detection of the wrist pulse through the proposed pulse taking platform has the same performance as that of the single sensor platform. 

**Figure 11:**
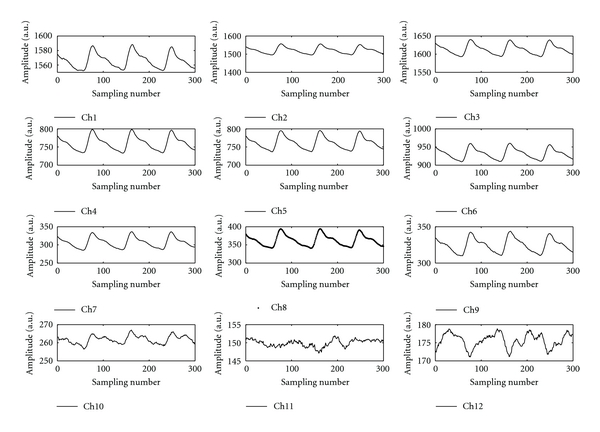
The waveform of each channel at Guan pulse taking position.

Additionally, the proposed pulse taking platform can measure twelve channels of wrist pulse simultaneously at Guan pulse-taking position. The waveform of each channel is displayed in Figure 11; it indicates 12 channels work well for the detection of radial artery signals. Since twelve-channel signals are acquired at Guan, the surface of the wrist pulse can be analyzed by using a surface fitting equation with core parameters of the pulse conditions, including PEAK, FERQ, LENGTH, WIDTH, AS, and DS as shown in Figure 8.

### 3.2. Results of the Proposed Pulse-Taking Platform in Clinic

The repeatability of the proposed pulse taking platform is listed in Table 1. The *t*-test results reveal that the *P* value is larger than  .05, which indicates that the means does not show significant differences between pre- and post-sampling during the 10-minute period.

To testify the differences among Fu, Zhong, and Chen pulse taking depths, an ANOVA test is implemented. After the ANOVA test, among all the parameters except FREQ, we observe significant differences (*P* < 0.05) in the mean values among the different pulse taking depths. The ANOVA results are tabulated in Table 2, in the form of mean ± SD. From the viewpoint of “means”, the differences of the parameters between Zhong and Chen pulse taking depths are smaller than those between the Fu and Zhong depths, as well as for the Fu and Chen pulse-taking depths. 

## 4. Discussion

### 4.1. Advantages of the Proposed Pulse Taking Platform

The wrist pulse waveforms are roughly divided into either a triple-humped wave, which has three peaks, or a double-humped wave, which has two peaks [17]. The proposed pulse taking platform can obtain the same type of pressure pulse waveform as shown in Figure 11. The strength, frequency, length, width, and trend of pulse conditions are simultaneously obtained from our designed pulse-taking platform. That means that the proposed pulse taking platform with the tactile sensor array satisfies the requirements of pulse diagnosis. Although the artery wrist is not a flat surface, the optimal pulse taking position focuses on the peak of the wrist pulse and the proposed pulse taking platform can be adjusted to the peak's occurrence at the nearby center of the tactile sensor array. In this way, the unique characteristics of wrist pulse signals similar to the single sensor can be obtained, and three-dimensional wrist pulse signals around the peak of wrist pulse can also be obtained. This pulse-taking method is consistent with the clinical method.

The main analytical methods for the single sensor are time domain and frequency domain. In the time domain, researchers have found unique characteristics of pulse waveforms such as a percussion wave, a tidal wave, and a dicrotic wave [16, 17]. Based on these characteristics, the pattern of pulse conditions can be classified. In addition, in the frequency domain, the distribution of spectrum and the resonance of wrist pulse signals have recently been investigated [18, 19]. Since the proposed platform can obtain the same pulse waveform as a single sensor, these analytical methods can equally apply to our recorded signals using the proposed pulse taking platform. 

It is valuable to investigate the length, width, and trend of pulse conditions. Up to now, these characteristics have not been easily detected by a single sensor [5]. To measure the width of the wrist pulses and the pressure pulse waveform, a combined detecting probe has been implemented by, for instance, Tyan et al. who proposed a pressure sensor for recording the pressure pulse waveform and a strain gauge for recording the width of wrist pulses [8]. However, it is easier to find these characteristics through our proposed platform with the sensor array, as shown in Figure 8. The length, width, and trend of wrist pulses obtained through three-dimensional wrist pulses are more compatible with clinical data, and the algorithm is easier than that of the single sensor.

Additionally, a pressure sensor is the best choice for imitating the pulse-taking feeling of a TCM doctor. A pressure sensor can be made by PVDF, PZT, Piezoresistor, and so on; while they can only detect dynamic characteristics of wrist pulses, they have limitations with regard to static characteristics of wrist pulses, especially in static pressure, which represents hold-down pressure [5]. The dynamic characteristics which represent the pulse waveform are compared at different pulse taking depth, such as Fu, Zhong, and Chen, and at different pulse taking positions, such as Cun, Guan, and Chi. The static characteristics represent the pulse waveform at specific pulse taking depth and static pressure at each pulse taking depth. According to the above definitions, the proposed platform can simultaneously detect dynamic characteristics and static characteristics during a single pulse-taking procedure, as shown in Figures 9 and 10, in which we can observe the change of the wrist pulse waveform and the change of hold-down pressure during the pulse taking procedure. Differential static pressure (namely, the change of hold-down pressure), such as *S*_21_ or *S*_32_ in Figure 9, may be useful to recognize the tension of wrist pulses in the future.

Conventionally, the optimal pulse taking position is at the maximum of the wrist pulse. Tyan et al. proposed a method to detect the optimal site for recording the pressure pulse waveform [8]. Since its detecting probe contained only single sensor, it made the pulse-taking procedure more complex. The sensing area of the sensor array is bigger than that of a single sensor. Hence, it is easy to find the peak location at the sensing area as shown in Figure 8. This characteristic should be beneficial with regard to developing an automatic pulse taking platform to find the optimal pulse taking position.

Table 3 lists the apparatus that have been reported in detecting wrist arterial pulse. Basically, single sensor cannot provide information enough to construct surface fitting for investigating the pulse length and pulse width. Although there are 9 probe-sensing elements in Tang's report, its arrangement, which is cruciform, limits the feasibility of surface fitting. On the other hand, static pressure represents the pulse taking pressure; it is an important index during pulse taking procedure. From the above comparison, the proposed pulse taking platform is suitable for imitating the procedure of taking pulses. 

To sum up, the proposed pulse-taking platform can provide not only dynamic characteristics as single sensor platform but also static pressure of taking pulse and shape parameters (pulse length and pulse width). Based on these characteristics, the trend parameters can be analyzed. And it is easier to find the optimal pulse taking position by the array sensor platform.

### 4.2. Verify Feasibility of the Proposed Pulse Taking Platform in Clinic

Two common acceptable principles of TCMPD are used to verify our assumption regarding the feasibility of quantifiable TCMPD researches with a tactile capacitive array sensor. One is that the pulse condition is almost not changed as long as no physical or psychological intervention exists in 10 minutes. The other is that the pulse conditions of the wrist radial artery are different among different pulse taking depths. These different pulses-taking depths in the terminology of TCMPD are called Fu, Zhong, and Chen.

One of the controversial issues of TCM pulse diagnosis is the repeatability of pulse conditions. This means that different doctors taking the pulse of the same subject may obtain different pulse conditions. According to the basic rule of pulse diagnosis, the pulse condition should not be changed in 10 minutes without any intervention. The result of the paired samples' *t*-test listed in Table 1 is consistent with clinic experiences. It shows that the repeatability of the proposed platform is feasible for quantification of TCMPD.

We evaluate the differences among Fu, Zhong, and Chen pulse taking depths with these defined core parameters. After an ANOVA analysis, the *P* value of STATIC, PEAK, FREQ, LENGTH, WIDTH, AS, and DS are  0.000,  0.000,  0.132,  0.000,  0.002,  0.000, and  0.000, respectively. More information is listed in Table 2. The results indicate that the frequency of the wrist pulse had no significant differences at the different pulse-taking depths; this means that once the wrist pulse is detected, the frequency of the wrist pulse is decided. This phenomenon corresponds with the clinic finding. On the other hand, other results, such as those for STATIC, PEAK, LENGTH, WIDTH, AS, and DS, have significant differences among different pulse-taking depths. Different combined parameters of pulse conditions at different pulse taking depths represent different health conditions. This quantified result indicates that the dynamic vertical characteristics of pulse conditions are different at different pulse taking depths. Furthermore, Jeon et al. proposed that the dynamic horizontal characteristics of pulse conditions are also different at different pulse taking positions, namely, Cun, Guan, and Chi [20]. This analysis methodology may be meaningful according to the dynamic vertical characteristics and dynamic horizontal characteristics of pulse conditions.

**Figure 12:**
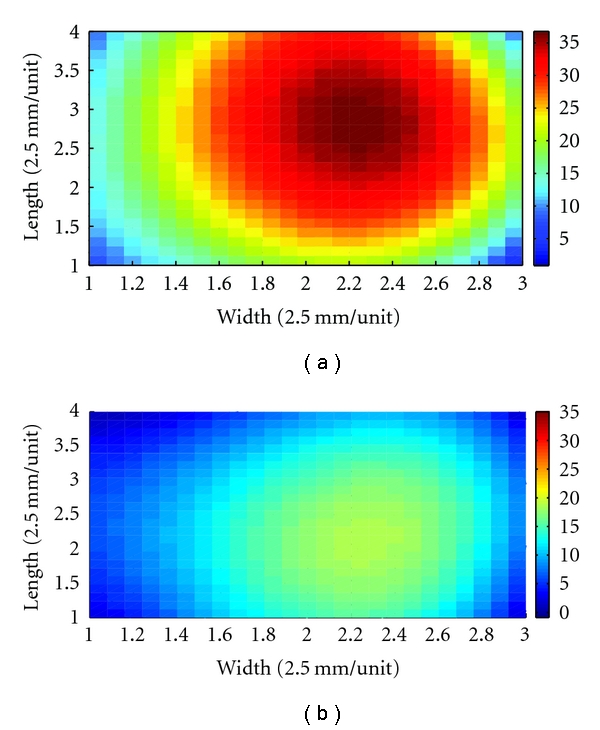
The characteristics of pulse conditions, such as strength, length, and width, are easily identified by 3D map. (a) The 3D map represents replete pulse. The strength, length, and width of pulse condition are higher and larger than (b). (b) The 3D map represents vacuous pulse.

The simple application was also proposed to explain the usefulness of proposed platform. The fingertip's feeling of replete pulse represents a general term for a pulse felt forceful at all the three sections, Cun, Guan, and Chi, also called forceful pulse. That means the response area of replete pulse is higher and larger at tactile array sensor and depicted in Figure 12(a). On the other hand, the response area of vacuous pulse is lower and smaller at tactile array sensor and depicted in Figure 12(b). According to this application, the strength, length, and width of pulse conditions are easily obtained from 3D map, as in Figure 12.

To sum up, the proposed pulse-taking platform is feasible for the quantification of TCMPD according both experiments, including repeated sampling wrist radial artery signals within 10 minutes and verification of difference of pulse conditions among different pulse-taking depths. This provides sufficient evidence to verify the basic theory of pulse diagnosis: the mapping relationship is meaningful between organs and pulse conditions. A more detailed mapping relationship will be checked in the future. Based on our results; therefore, we infer that the proposed pulse-taking platform with an array sensor is feasible in pulse diagnosis.

## 5. Conclusions

 The aim of this proposal is to provide an innovative method to obtain full information for wrist pulse signals, such as temporal and spatial properties. A pulse taking platform with an array sensor is implemented to carry out this purpose. The length, width, and trend of pulse conditions can be easily detected by our proposed platform, and the results reveal that the performance of the pulse-taking platform with an array sensor is better than that of a single sensor, since the proposed platform obtains not only the unique characteristic waveform but also the surface of the wrist pressure waveform. The paired samples' *t*-test shows that this proposed platform is feasible to repeat the pulse taking procedure, and the results of the ANOVA test for the pulse taking depths proves the array sensor pulse taking platform is practicable for quantified research of TCMPD. In the future, using this platform to evaluate the basic principle of TCM will open a new quantitative method for TCM.

## Figures and Tables

**Table 1:** The paired samples *t*-test results.

Item of paired samples	Sampling procedure	Mean	SD	*t*-value	*P* value
STATIC	Pre-sampling	3.4164E5	5.8371E4	1.030	0.310
	Post-sampling	3.6327E5	1.2160E5		
PEAK	Pre-sampling	17420.10	2344.340	0.289	0.775
	Post-sampling	17256.60	1793.196		
PERIOD	Pre-sampling	74.25	4.610	1.716	0.103
	Post-sampling	72.95	3.649		
LENGTH	Pre-sampling	0.434375	0.1305904	1.610	0.124
	Post-sampling	0.396875	0.1625696		
WIDTH	Pre-sampling	0.604565	0.2270977	1.288	0.213
	Post-sampling	0.540920	0.2613039		
AS	Pre-sampling	2939.438878	3325.29	−1.846	0.082
	Post-sampling	4058.655372	4455.78		
DS	Pre-sampling	−2896.963287	2875.43	1.361	0.195
	Post-sampling	−4735.477193	6143.07		

**Table 2:** ANOVA of measurements.

	Fu	Zhong	Chen	*P* value
STATIC	802.75 ± 362.91	1283.10 ± 397.50	1693.26 ± 411.73	0.000*
PEAK	15.23 ± 14.84	35.73 ± 16.93	37.05 ± 13.73	0.000*
PERIOD	72.590 ± 8.12	70.48 ± 7.42	71.30 ± 7.29	0.132
LENGTH	0.17 ± 0.15	0.32 ± 0.14	0.37 ± 0.09	0.000*
WIDTH	0.26 ± 0.24	0.37 ± 0.24	0.34 ± 0.20	0.002*
AS	6.90 ± 7.28	11.13 ± 6.81	10.14 ± 8.88	0.000*
DS	−1.34 ± 3.25	−4.86 ± 6.88	−8.14 ± 6.12	0.000*

*The mean difference is significant at the  0.05 level.

**Table 3:** The performance comparison in the literatures.

Researcher	Length sensing element	Width sensing element	Probe sensing element	Static Pressure	Surface analysis
Tyan et al. [8]	1	1	1	○	X
Tang and Sun [11]	3	7	9	○	X
Jim et al. [13]	2	2	4	X	X
Our proposed	4	3	12	○	○
